# Presence of novel triple mutations in the *pvdhfr* from *Plasmodium vivax* in Mangaluru city area in the southwestern coastal region of India

**DOI:** 10.1186/s12936-018-2316-3

**Published:** 2018-04-16

**Authors:** Shiny Joy, Susanta K. Ghosh, Rajeshwara N. Achur, D. Channe Gowda, Namita Surolia

**Affiliations:** 1 Molecular Biology and Genetics Unit, Jawaharlal Nehru Centre for Advanced Scientific Research, Jakkur, Bangalore, India; 2 Department of Biological Control, National Institute of Malaria Research, Poojanahalli, Bangalore, India; 3 Department of Biochemistry, Kuvempu University, Shankaraghatta, Shivamogga District, Karnataka India; 4 Department of Biochemistry and Molecular Biology, The Pennsylvania State University College of Medicine, 500 University Drive, Hershey, PA 17033 USA

**Keywords:** Dihydrofolate reductase, Dihydropteroate synthase, Single nucleotide polymorphisms, Restriction fragment length polymorphisms, Sulfadoxine/pyrimethamine, Mangaluru, India

## Abstract

**Background:**

Genes encoding dihydrofolate reductase (*dhfr*) and dihydropteroate synthase (*dhps*) are the targets of sulfadoxine–pyrimethamine (SP) present in artemisinin based combination therapy (ACT; artesunate + sulfadoxine pyrimethamine) for *Plasmodium falciparum*. Although SP is generally not used to treat vivax infection, mutations in *dhfr* and *dhps* that confer antifolate resistance in *Plasmodium vivax* are common; which may be attributed to its sympatric existence with *P. falciparum*. Current study was aimed to determine the pattern of mutations in *dhfr* and *dhps* in *P. vivax* isolates from Mangaluru region.

**Methods:**

A total of 140 blood samples were collected from *P. vivax*-infected people attending Wenlock Hospital Mangaluru during July 2014 to January 2016. Out of 140 isolates, 25 (18%) and 50 (36%) isolates were selected randomly for sequence analysis of *pvdhfr* and *pvdhps* genes respectively. Fragment of *pvdhps* and full length *pvdhfr* were amplified, sequenced and analysed for single nucleotide polymorphisms. *dhps* was analysed by PCR–RFLP also, to detect the two specific mutations (A383G and A553G).

**Results:**

Analysis of *pvdhps* sequences from 50 isolates revealed single and double mutants at 38 and 46% respectively. Three non-synonymous mutations (K55R, S58R and S117N) were identified for *pvdhfr.* Among these, K55R was detected for the first time.

**Conclusions:**

The current study indicates that *P. vivax dhps* and *dhfr* mutant alleles are prevalent in this area, suggesting significant SP pressure.

**Electronic supplementary material:**

The online version of this article (10.1186/s12936-018-2316-3) contains supplementary material, which is available to authorized users.

## Background

Anti-malarial drug resistance is a major public health issue in many countries. After the emergence of chloroquine resistance in *Plasmodium falciparum*, sulfadoxine–pyrimethamine (SP) drug combination is used widely especially in mixed infection cases. In both *P. falciparum* and *Plasmodium vivax*, combination of antifolate drugs target the folate metabolism by acting on two enzymes, dihydrofolate reductase (*dhfr*) and dihydropteroate synthase (*dhps*). Mutations in these genes lead to SP resistance. Several mutations have been identified in *P. vivax dhfr*. Of these, mutations at codon 57, 58, 61, 117 and 173 are known to be associated with pyrimethamine resistance [1]. Similarly five point mutations at codon 382, 383, 512, 553 and 585 of *P. vivax dhps* are involved in sulfadoxine resistance. Of these, mutations at codons 383 and 553 (S_382_G_383_K_512_G_553_V_585_ genotype) were highly correlated with sulfadoxine resistance [2].

In India, *P. vivax* accounts for more than half of the malaria cases (53%) [3]. Though some clinical cases of resistance have been reported [4–7], CQ remains the drug regimen for vivax malaria. Artemisinin-based combination therapy (ACT) is used for the treatment of mixed infection and complicated *P. vivax* cases [8]. Thus, analysis of *pvdhfr* and *pvdhps* gene sequences predict the SP efficacy in a particular region.

Mangaluru, the coastal city of Karnataka, has a tropical climate, with over 6% mixed infection cases [9]. Although SP treatment is not recommended for *P. vivax* infections in this area, parasites get exposed to this drug when SP is given to *P. falciparum* and *P. vivax* mixed infection cases. The current study was aimed to analyse the polymorphisms in *dhfr* and *dhps* genes of *P. vivax* in this area.

## Methods

### Sample collection and ethics

The study was conducted at Wenlock District Hospital, Mangaluru city, located at the Southwestern coastal India in the Dakshina Kannada district in Karnataka state, India. Samples were collected from June 2014 to December 2015 after obtaining informed consents. A total of 140 mono-infection *P. vivax* positive patients were collected. Ethical clearance was obtained for all the patients. Out of 140 isolates, 25 (18%) and 50 (36%) isolates were selected randomly and included for the study. After confirming infections by expert microscopic examinations of Giemsa-stained thick and thin blood smears, and reconfirmed with bivalent rapid diagnostic test kit (Falcivax^®^ from tulip, Goa), blood samples were collected on Whatman 3MM filter paper. Blood spots on filter papers were allowed to air-dry and then placed individually in plastic bags and stored at − 20 °C until processed. The study was approved by the Institutional Review Boards (IRB) of The Pennsylvania State University College of Medicine, Hershey, PA, USA, by incorporating approval from the ethics committee of Kuvempu University, Shivamogga, Karnataka, India. The IRB approved Protocol Number is 34148. The study designed was according to the NIH and ICMR ethical guidelines.

### DNA extraction and species confirmation

Genomic DNA was extracted from filter paper spots as described earlier [10]. The extract was stored at − 20 °C until used. Amplification of SP resistance associated genes; the *P. vivax dhfr* and *dhps* genes from 25 to 50 isolates were amplified using primers and conditions as described earlier [2, 11]. For *dhfr* amplification, PCR conditions used were initial denaturation at 95 °C/30 s, denaturation at 95 °C/30 s, annealing at 62 °C/30 s and extension at 68 °C/60 s for 30 cycle and final extension at 68 °C/5 min. For *dhps*, nested PCR strategy was used as described earlier [2]. Restriction digestion of *dhps*: to detect the mutations at 383 and 553, Msp1 and Msc1 restriction enzymes were used respectively. Restriction digestion was carried out by using previously described PCR–RFLP method [2]. The mutation at codon 383 was detected by restriction digestion with Msp1 enzyme. If the mutation is present at 383 codon (383 Gly), Msp1 cleaves the 703 bp fragment to 655 bp and 48 bp. The mutation at 553 was detected by restriction digestion with Msc1 enzyme and PCR product of 170 bp. If the mutation is present at codon 553 (553 Gly), Msc1 does not digest 170 bp fragment, but it cleaves the 170 bp fragment to 143 and 28 bp fragment if no mutation is present. The DNA fragments obtained after RFLP analysis were electrophoresed on 1 and 3% agarose gels (Additional file 1: Figure S1).

### Sequence analysis

Sequencing was performed on purified product of 711 bp of *pvdhfr* and 705 bp of *pvdhps*. The PCR products were extracted from gels using Gel Extraction kit (Sigma-Aldrich, St Louis, MO, USA) and sequenced. Sequencing of genes from each isolate was performed on an ABI Prism 377 DNA Sequencer equipped with semi adaptive version 3.0. Nucleotide sequences were analysed using blast and Bio Edit Sequence Alignment Editor and compared with reference sequences of Gen-Bank Accession Numbers X98123 and AY186730 for *pvdhfr* and *pvdhps* respectively. All PCR amplifications were carried out with a negative control (no template) and polymorphisms in these two genes were confirmed by reading both the forward and reverse strands.

## Results

### *Plasmodium vivax dhfr* and *pvdhps* gene polymorphisms

#### dhfr

Complete coding region of *dhfr* from 25 isolates was amplified and sequenced. On comparison with reference strain, three different point mutations (K55R, S58R and S117N) were observed. Novel mutation (K55R) was observed in 36% of the samples. Mutations S58R and S117N were observed in all the samples (100%). Frequency distribution of mutation at each codon and haplotype distribution in *dhfr* are given in Table 1. Type 2 tandem repeat variation was observed in all the samples.

**Table 1:** Frequency distribution of mutation at each codon and haplotype in *pvdhfr* gene

No	Aminoacid change	Isolates number (%)
1.	K55R (AAG to AGG)	9 (36)
2.	S58R (AGC to AGG)	25 (100)
3.	S117N (AGC to AAC)	25 (100)
Haplotype		
1.	No mutation (Wild type)	0 (0)
2.	Double mutant (58R117N)	16 (64)
3.	Triple mutant (55R58R117N)	9 (36)

Underlined font represents base change

#### dhps

For *dhps* a total of 50 isolates were amplified and sequenced. Sequence analysis revealed two mutations A383G and A553G in different frequencies. Mutations at each codon and haplotype frequencies are shown in Table 2. Three different genotypes, 16% of wild type (SAKAV), 38% of single mutant (SGKAV) and 46% of double mutant (SGKGV) were observed. Both sequencing and PCR–RFLP was done for majority of the isolates and revealed same result. None of the additional mutations identified by sequencing.

**Table 2:** Frequency distribution of mutation at each codon and haplotype in *pvdhps* gene

No	Aminoacid change	Isolates number (%)
1.	A383G (GCC to GGC)	42 (84)
2.	A553G (GCC to GGC)	23 (46)
Haplotype		
1.	No mutation (wild type)	8 (16)
2.	Single mutant (383G)	19 (38)
3.	Double mutant (383G553G)	23 (46)

## Discussion

Polymorphisms in two of the folate biosynthesis pathway genes *dhps* and *dhfr* contribute to sulfadoxine and pyrimethamine drug resistance respectively in *P. falciparum*. Similar drug resistance mechanisms are present in *P. vivax* due to the conserved nature of the homologs of these two enzymes [12]. Mutations at five positions in *P. vivax* DHFR–thymidylate synthase gene (A15, N50, R58, N117, and I173) as determined by the secondary structure analysis are identified corresponding to 16, 51, 59, 108, and 164 respectively in *P. falciparum*. The *pfdhfr* primary mutation (S108N) combined with secondary mutations at codons 50, 51, 59, and 164 lead to enhanced pyrimethamine resistance [13]. Similarly, mutations at codons 436, 437, 540, 581, and 613 of *dhps* lead to sulfadoxine resistance in *P. falciparum* [14]. Five mutations at homologous gene *pvdhps* have been identified at codons 382, 383, 512, 553 and 585. Among these, mutations at codon 383 and 553 are solely responsible for sulfadoxine resistance. Additional mutations confer higher levels of resistance [2, 15].

The current study reports the antifolate resistance in *P. vivax* infection, analysing *dhfr* and *dhps* mutations in isolates collected from Mangaluru and its surrounding area. Analysis of point mutations in *dhfr* among isolates from Indian sub-continent earlier revealed presence of four distinct genotypes: the wild, single mutant, double mutant, and quadruple mutant [16]. The current study reveals occurrence of only double and triple mutants, while none of the isolates are found to contain single or quadruple *dhfr* mutations. No isolates carrying wild type allele and mutations at amino acids 58 and 117 of *dhfr* were detected. The frequency of isolates carrying double mutations was found to be the highest as reported earlier from Chennai region from South India [16]. These mutations (in combination) are usually associated with resistance, due to slow clearance after SP treatment [17].

A novel mutation K55R is observed together with S58R and S117N accounting to 36% triple mutants. Presence of this haplotype consisting of these mutants is observed for the first time. *dhfr* sequences from all the isolates revealed type 2 tandem repeat variants similar to previous observations reported from India [18, 19]. Type 1 tandem repeat variant was found to be associated with quadruple mutations and higher levels of resistance which can serve as a molecular marker to predict the risk of mutations in any geographical area of Indian subcontinent [16]. Since the presence of double mutations and type 2 tandem repeat variants are predominant in our study, the level of resistance may not be that high, however continuous monitoring and surveillance is essential to predict the emergence of drug resistance in this area. As previous studies have reported, prevalence of *dhfr* mutant alleles are seen in the areas experiencing higher levels of SP pressure while wild type *dhfr* genotype is maintained in the areas with no or little SP pressure [1, 18]. The presence of mutations in the current study is indicative of SP pressure in the area.

*Plasmodium vivax dhps* sequences from 50 isolates were analysed. Two resistance conferring mutations A383G and A553G are observed in isolates with 84 and 46% respectively. These SNPs attribute to increased sulfadoxine resistance levels [12]. Wild type *dhps* sequences (SAKAV genotype) were observed only in 16% isolates while single mutants (SGKAV) at 38% and double mutants (SGKGV) at 46% are observed. PCR–RFLP results corroborated similar trend. No additional mutations were detected by direct sequencing. Evaluation of these two markers (*dhfr* and *dhps*) are important to predict the antifolate drug resistance in this area and this calls for continuous monitoring for deciding anti-malarial drug policy in the region.

## Conclusions

The results indicate that since the isolates from this area are exposed to antifolate drugs they have developed mutations in their *dhfr* and *dhps* genes.

## Additional file

**Additional file 1: Figure S1.** PCR–RFLP of the *pvdhps* gene.

## Abbreviations

SP: sulfadoxine–pyrimethamine
SNPs: single nucleotide polymorphisms
ACT: artemisinin-based combination therapy
CQ: chloroquine
RDT: rapid diagnostic test
PCR–RFLP: polymerase chain reaction–restriction fragment length polymorphisms
ICMR: Indian Council of Medical Research
