# Supplementary material for: Presence of novel triple mutations in the pvdhfr from Plasmodium vivax in Mangaluru city area in the southwestern coastal region of India
Source: Malar J. 2018 Apr 16;17:167. doi: 10.1186/s12936-018-2316-3 (PMC5902849; doi:10.1186/s12936-018-2316-3)
Supplement: Supplementary file 1 — Additional file 1: Figure S1. PCR–RFLP of the pvdhps gene. [file 12936_2018_2316_MOESM1_ESM.pptx]

## Slide 1
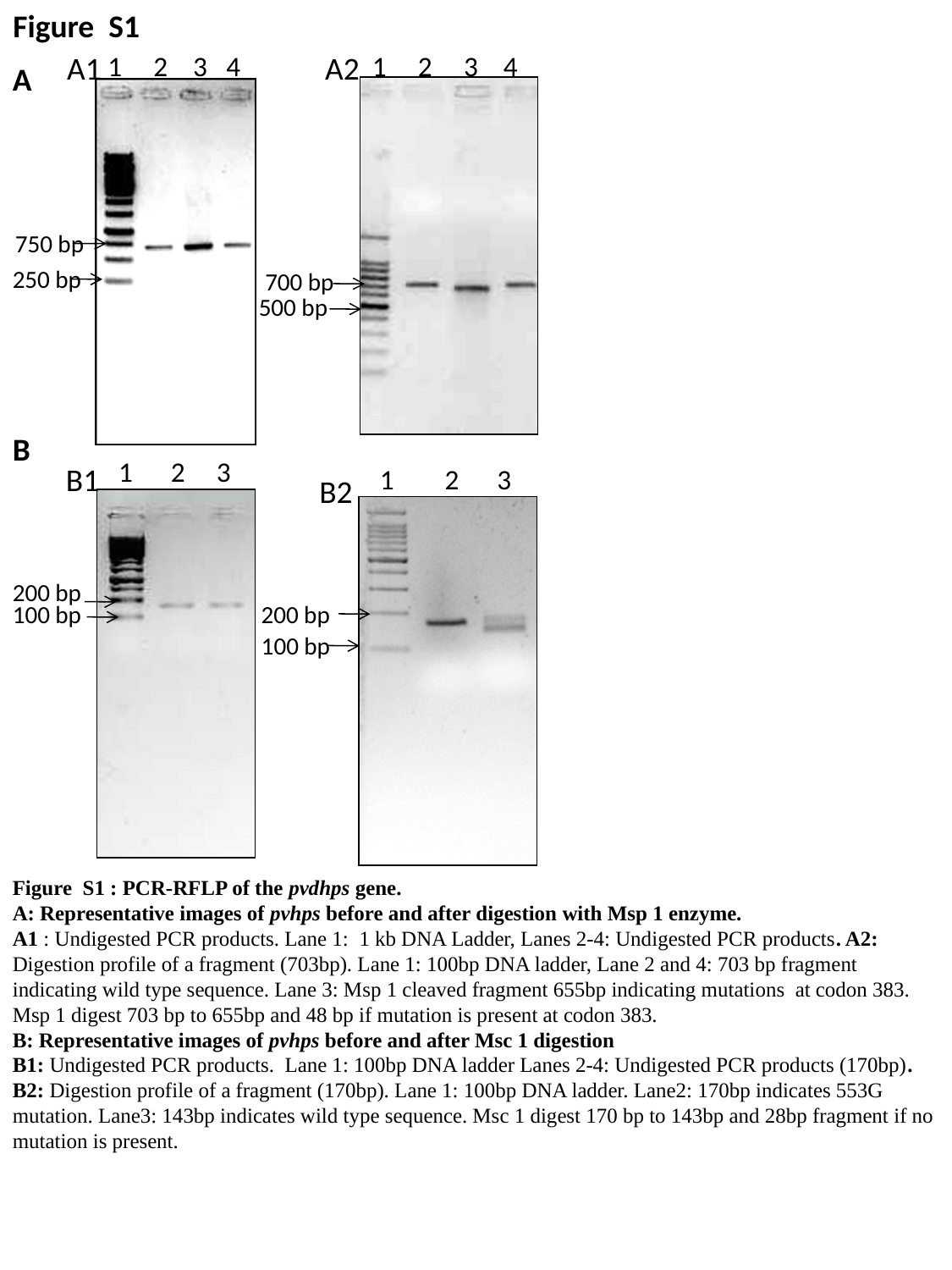

Figure S1
1 2 3 4
750 bp
250 bp
A1
A2
1 2 3 4
700 bp
500 bp
A
B
 1 2 3
B1
200 bp
100 bp
1 2 3
B2
200 bp
 100 bp
Figure S1 : PCR-RFLP of the pvdhps gene.
A: Representative images of pvhps before and after digestion with Msp 1 enzyme.
A1 : Undigested PCR products. Lane 1: 1 kb DNA Ladder, Lanes 2-4: Undigested PCR products. A2: Digestion profile of a fragment (703bp). Lane 1: 100bp DNA ladder, Lane 2 and 4: 703 bp fragment indicating wild type sequence. Lane 3: Msp 1 cleaved fragment 655bp indicating mutations at codon 383. Msp 1 digest 703 bp to 655bp and 48 bp if mutation is present at codon 383.
B: Representative images of pvhps before and after Msc 1 digestion
B1: Undigested PCR products. Lane 1: 100bp DNA ladder Lanes 2-4: Undigested PCR products (170bp). B2: Digestion profile of a fragment (170bp). Lane 1: 100bp DNA ladder. Lane2: 170bp indicates 553G mutation. Lane3: 143bp indicates wild type sequence. Msc 1 digest 170 bp to 143bp and 28bp fragment if no mutation is present.
